# Individual HIV Risk versus Population Impact of Risk Compensation after HIV Preexposure Prophylaxis Initiation among Men Who Have Sex with Men

**DOI:** 10.1371/journal.pone.0169484

**Published:** 2017-01-06

**Authors:** Samuel M. Jenness, Akshay Sharma, Steven M. Goodreau, Eli S. Rosenberg, Kevin M. Weiss, Karen W. Hoover, Dawn K. Smith, Patrick Sullivan

**Affiliations:** 1 Department of Epidemiology, Emory University, Atlanta, Georgia, United States of America; 2 Department of Anthropology, University of Washington, Seattle, Washington, United States of America; 3 Division of HIV/AIDS Prevention, Centers for Disease Control and Prevention, Atlanta, Georgia, United States of America; 4 Department of Global Health, Emory University, Atlanta, Georgia, United States of America; University of New South Wales, AUSTRALIA

## Abstract

**Objectives:**

Risk compensation (RC) could reduce or offset the biological prevention benefits of HIV preexposure prophylaxis (PrEP) among those at substantial risk of infection, including men who have sex with men (MSM). We investigated the potential extent and causal mechanisms through which RC could impact HIV transmission at the population and individual levels.

**Methods:**

Using a stochastic network-based mathematical model of HIV transmission dynamics among MSM in the United States, we simulated RC as a reduction in the probability of condom use after initiating PrEP, with heterogeneity by PrEP adherence profiles and partnership type in which RC occurred. Outcomes were changes to population-level HIV incidence and individual-level acquisition risk.

**Results:**

When RC was limited to MSM highly/moderately adherent to PrEP, 100% RC (full replacement of condoms) resulted in a 2% relative decline in incidence compared to no RC, but an 8% relative increase in infection risk for MSM on PrEP. This resulted from confounding by indication: RC increased the number of MSM indicated for PrEP as a function of more condomless anal intercourse among men otherwise not indicated for PrEP; this led to an increased PrEP uptake and subsequent decline in incidence.

**Conclusions:**

RC is unlikely to decrease the prevention impact of PrEP, and in some cases RC may be counterintuitively beneficial at the population level. This depended on PrEP uptake scaling with behavioral indications. Due to the increased acquisition risk associated with RC, however, clinicians should continue to support PrEP as a supplement rather than replacement of condoms.

## Introduction

Preexposure prophylaxis (PrEP) has been shown to be highly effective in preventing infection with the human immunodeficiency virus (HIV) among persons with sexual behaviors that place them at substantial risk, including men who have sex with men (MSM) [1]. In the United States, MSM have experienced the highest HIV incidence of any risk group, accounting for 67% of new HIV cases but only 4% of the adult male population [2]. Following the completion of clinical trials testing the efficacy of the oral daily tenofovir disoproxil fumarate and emtricitabine formulation as PrEP [3], research priorities have begun to address questions on how to scale-up PrEP to the broader at-risk population and the long-term implications of its use in the context of male-to-male sexual partnerships [4].

One public health concern has been whether PrEP will replace rather than supplement existing HIV prevention approaches such as condoms [5]. Although condoms are effective at preventing HIV transmission [6,7], the rates of condomless anal intercourse (CAI) among MSM have been increasing since the early 2000s, before PrEP became available [8], and could rise further with increased uptake of PrEP [9]. Risk compensation, broadly defined, is an intentional increase in risk behaviors following the initiation of disease prevention modalities known to be effective. This phenomenon could reduce the population-level prevention benefits of PrEP, especially if it occurs among persons who are not fully adherent to the recommended daily dosing associated with high levels of PrEP effectiveness [10]. Risk compensation could also modify the efficiency, and therefore cost-effectiveness, of PrEP since its pharmacological efficacy to prevent HIV could be offset by higher infection risk such that the number needed to treat to avert one infection could increase [11].

Open-label PrEP demonstration studies that have tracked outcomes before and after initiation of PrEP have found minimal evidence of risk compensation, as measured by both self-reported risk behaviors and non-HIV sexually transmitted infection (STI) incidence [10,12,13]. Where there has been evidence of risk compensation in MSM populations, it is largely limited to reduced condom use rather than other behaviors like acquiring greater number of sexual partners [14,15]. Evaluating risk compensation within these studies, however, may not provide adequate evidence to infer long-term sexual behavior patterns of MSM on PrEP outside of research settings for at least two reasons. First, study eligibility criteria typically screen out low-risk persons in order to achieve statistical power for measuring differences in incident HIV infections, resulting in a sample of higher-risk persons for whom sexual behavior may have plateaued even before starting PrEP. Second, follow-up time in some studies may be too short to quantify long-term outcomes, or differential changes in behavior by study arm. Recent data from the longest-running open-label PrEP study of MSM in the United Kingdom suggest that risk compensation occurred among some PrEP recipients, with a larger proportion of MSM on the immediate PrEP arm (21%) compared to a deferred arm (12%) reporting CAI [16]. Demonstration studies have also not yet examined the interaction between risk compensation and PrEP adherence levels or partnership types within which CAI occurs. If compensation were limited to MSM who were highly adherent to PrEP or had monogamous main partnerships only, the overall impact of risk compensation on the prevention effectiveness of PrEP could be small.

In this study, we use mathematical modeling to estimate the potential effects of condom-related risk compensation following initiation of PrEP among MSM in the US. Building on an earlier model [17] in which we predicted the epidemiological impact of US Public Health Service/Centers for Disease Control and Prevention (CDC) clinical practice guidelines for PrEP [18], our current goal is to investigate the interaction between risk compensation level, PrEP adherence profile, and MSM partnership types within which condom-related behavioral change may occur following PrEP initiation. Our modeling approach allows for a comparison between population-level HIV incidence and individual-level HIV acquisition risks associated with risk compensation that may emerge during the long-term utilization of PrEP.

## Methods

### Model Framework

This study extends our stochastic agent-based model of HIV transmission dynamics over sexual partnership networks, originally developed to investigate the role of PrEP on HIV incidence among MSM in the United States [17]. Our model was programmed using the *EpiModel* software platform (http://epimodel.org/), which depends on the statistical framework of temporal exponential random graph models (ERGMs) to simulate highly structured sexual networks of MSM partnerships over time. Most behavioral data to parameterize the model were derived from an HIV incidence cohort and a cross-sectional sexual network study of MSM in Atlanta [19,20]. This current model uses the same parameterization for the behavioral and clinical components as our earlier modeling study [17]. Partnerships were divided into main, casual, and one-time types, distinguished by their average duration and predictors of formation, including the number of current ongoing partnerships (network degree), the difference in age between the two partnerships (age homophily), and sorting by sexual position.

For interhost and intrahost epidemiology, the HIV clinical model simulated all aspects of the natural progression of HIV after infection, with a representation of HIV viral load, HIV testing, antiretroviral therapy (ART) initiation and adherence profiles, and eventual disease-induced mortality for MSM with treatment failure. Within HIV-discordant partners, predictors of HIV transmission during anal intercourse (AI) included viral load [21], condom use [7], receptive versus insertive sexual position [22], circumcision for the insertive negative partner [23], and the presence of the CCR5-Δ32 genetic allele [24,25].

### PrEP Indications, Uptake, and Discontinuation

PrEP indications were modeled based on the CDC’s guidelines for clinical practice for MSM [18]. Men were simulated to test for HIV at approximately yearly intervals, based on empirical HIV testing frequency data [19], at which point they were assessed for the three behavioral conditions indicating PrEP in the guidelines: condomless anal intercourse (CAI) in a status-unknown monogamous partnership, CAI outside of a monogamous partnership, and any AI in a known HIV-discordant partnership. Behavior was tracked over the six months prior to that assessment (the “risk window”), and any conditions accumulated over the window to create an indication for PrEP. The union of these three behavioral conditions defined an indication for PrEP according to the CDC guidelines [26].

PrEP uptake was modeled, following earlier approaches [17], based on a fixed coverage proportion (coverage = number currently using PrEP / number currently indicated to use PrEP). At each time step during the intervention simulations, up to 40% of men with indications were allowed on PrEP. We chose this threshold level based on recent behavioral research estimating that 61% of MSM had interest in taking PrEP [27], which we conservatively reduced to 40% to account for the probable drop-off between stated interest and actual use. This coverage level was also consistent with our past PrEP modeling activities [17,28]. When the coverage threshold was reached, additional men were not started on PrEP until other men discontinued PrEP, causing coverage to fall below the threshold. The coverage threshold was fixed as a proportion at 40% but the total number of men on PrEP could grow if the number of behaviorally indicated men (the denominator in the coverage equation above) increased. Less than 100% coverage of indicated men implicitly represents lack of PrEP awareness, interest, and access all affecting imperfect uptake of PrEP even though these were not explicitly simulated in our model [29]. PrEP uptake conditional on indications was homogeneous by demographic and behavioral attributes.

We assigned PrEP medication adherence levels based on empirical data from a three-city PrEP demonstration project: 21.1% of men were categorized as non-adherent (0 pills/week), 7.0% as taking <2 pills/week (low adherence), 10.0% at 2–3 pills/week (moderate adherence), and 61.9% at 4+ pills/week (high adherence) [12]. Use of PrEP resulted in a reduction of the per-act probability of infection that was correlated with adherence level: 0%, 31%, 81%, and 95%, respectively for non-adherent to high-adherence groups [10].

Men discontinued PrEP also based on the CDC guidelines, which recommend reassessment of risk behavior every 12 months and potential cessation of prescriptions if no longer indicated. In our model, we replicated this by reevaluating the dynamic risk behavior of each man on PrEP at yearly intervals after PrEP initiation; if men no longer exhibited PrEP indications within the 6-month risk window, then they stopped PrEP. Men who stopped were allowed to restart PrEP if they exhibited indications at their next HIV diagnostic visit and the coverage threshold had not been exceeded.

### Risk Compensation

Risk compensation was modeled as the reduction in the probability of condom use at each sexual act. Condom-related risk compensation has been more common among MSM than other forms of behavioral change, such as increases in the number of partners [14,15]. Base per-act condom use probabilities before compensation were parameterized from empirical data [19], and depended on partnership type (main, casual, versus one-off) and included a fixed class attribute to allow for consistent use of condoms among a subset of MSM. We modeled condom-related risk compensation along a gradient from 0% to 100%, where 0% was no reduction in condom use and 100% was full elimination of condoms during AI. In the absence of partnership-level behavioral data on PrEP, we assumed that the choice to use condoms was driven by the PrEP user in the partnership, rather than by a dyadic decision-making process.

Our main sensitivity analyses varied the level of risk compensation across different PrEP adherence profiles and partnership types. We hypothesized that risk compensation, if it were to occur, would be most likely among highly adherent PrEP users and least likely among the non-adherent. Therefore, we modeled risk compensation along the same 0% to 100% gradient assuming that it occurred in increasingly wider subsets of PrEP users: high adherence only; high and moderate adherence; high, moderate, and low adherence; and high, moderate, low, and non-adherent. Secondary analyses explored compensation along the same gradient, but limited to main partnerships or known HIV-discordant partnerships; we hypothesized that these were more likely candidates than casual or unknown-status partnerships for risk compensation to occur.

### Model Outcomes and Statistical Analysis

Because risk compensation could potentially impact both population-level epidemiology and individual-level HIV infection risk, our analyses considered both outcomes. Epidemiological outcomes tracked were the HIV incidence rate in each scenario, the percent of infections averted (PIA) compared to a scenario in which no PrEP was provided, and the number needed to treat (NNT) with one year of PrEP to prevent one new HIV infection. Individual-level outcomes included the expected number of infection events per 1000 exposures overall and by current PrEP status. To quantify use of PrEP in the full population, including men who were not indicated for PrEP, we calculated the total weeks on PrEP per 100 weeks of susceptible (HIV-uninfected) person-time. Although the coverage proportion remained constant at the 40% threshold, this person-time measure could increase if the total number of PrEP-indicated MSM increased in the population. Due to the stochastic nature of the models, we simulated each scenario 250 times, and reported means and 95% credible intervals (the middle 95% of each set of 250 observations) from across the simulations for each statistic.

The software code for reproducing model simulations and data analyses may be found at *http*:*//github*.*com/statnet/HIV-Risk-Comp/*.

## Results

**Fig 1:**
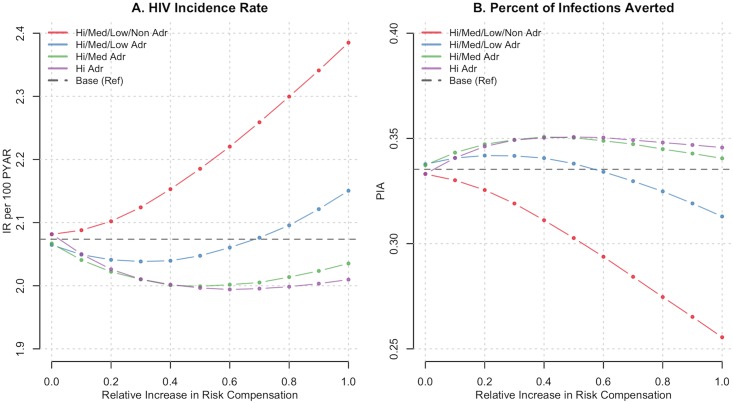
The HIV incidence rate per 100 person-years at risk (Panel A) and percent of infections averted relative to a scenario in which no PrEP was used (Panel B), by the interaction of relative levels of condom-related risk compensation (RC) and PrEP adherence profile in which compensation occurred. The red line depicts RC in all four PrEP medication adherence profiles (high, medium, low, and non-adherent), the blue in the top three (high, medium, low) green in the top two (high and medium) and purple in the high only. The horizontal dashed lines shows a comparison of the two outcomes (incidence and PIA) if RC were not to occur (0% RC). All points summarize the empirical distribution of 250 simulations of each scenario.

Table 1 and Fig 1 show the population-level epidemiological results. In the base scenario in which there was no risk compensation, the HIV incidence rate was 2.08 per 100 person-years at risk. Implementing PrEP according to CDC guidelines would avert 33.3% of expected cases over the next 10 years, assuming that no risk compensation were to occur. When partial (50%) or complete (100%) risk compensation occurred and was limited to MSM who were highly or moderately adherent to PrEP, the PIA increased slightly to between 34–35% (Table 1). When risk compensation occurred among those with high, moderate, or low adherence, the HIV incidence rate declined from the base scenario until compensation reached a threshold level of 60%, at which point it increased above base level (Fig 1A). The mechanisms for this counterintuitive result are described below.

**Table 1:** HIV Incidence Rate, Percent of Infections Averted, and Number Needed to Treat by Risk Compensation Level, by PrEP Adherence Level and Partnership Types, among US MSM.

Model Scenario	Incidence Rate^1^	PIA^3^	NNT^4^
	*(95% CrI)*^2^	*(95% CrI)*	*(95% CrI)*
**Base Scenario**			
0% RC	2.08 (0.18, 4.51)	33.3 (29.1, 37.3)	24 (21, 28)
**Adherence Groups (All Partnership Types)**			
* High*			
50% RC	1.99 (0.10, 4.42)	35.1 (31.3, 39.1)	26 (23, 29)
100% RC	2.00 (0.13, 4.43)	34.6 (30.1, 38.3)	27 (24, 31)
* High*, *Moderate*			
50% RC	2.01 (0.11, 4.40)	34.9 (31.4, 38.7)	26 (23, 29)
100% RC	2.04 (0.19, 4.50)	33.9 (30.3, 38.1)	28 (24, 31)
* High*, *Moderate*, *Low*			
50% RC	2.03 (0.15, 4.48)	34.0 (30.0, 38.3)	27 (24, 31)
100% RC	2.12 (0.19, 4.62)	32.0 (28.1, 35.9)	29 (26, 34)
* High*, *Moderate*, *Low*, *Non-Adherent*			
50% RC	2.19 (0.19, 4.62)	30.4 (26.3, 34.5)	31 (27, 36)
100% RC	2.39 (0.42, 5.11)	25.4 (21.2, 29.9)	38 (32, 47)
**Partnership Type (All Adherence Groups)**			
* Main Partners Only*			
50% RC	2.14 (0.26, 4.64)	32.1 (28.4, 35.6)	25 (23, 29)
100% RC	2.16 (0.29, 4.66)	31.2 (27.2, 34.5)	26 (23, 30)
* Known HIV-discordant Partners Only*			
50% RC	2.19 (0.28, 4.71)	30.7 (27.1, 34.6)	27 (23, 31)
100% RC	2.30 (0.40, 4.89)	28.0 (23.8, 32.5)	30 (25, 35)

^1^ Incidence per 100 person-years at risk

^2^ 95% CrI = 95% credible interval from the simulations

^3^ Percent of infections averted compared to expected in No PrEP scenario

^4^ Number needed to treat on PrEP for one year to avert one new infection

HIV incidence rates increased and the PIA decreased at all levels of risk compensation when it occurred among all four adherence groups, including MSM who were completely non-adherent to their prescriptions (Fig 1A and 1B). When risk compensation was allowed in all four adherence groups but limited to main or known HIV-discordant partners only, HIV incidence rates monotonically increased as compensation increased, although to a lesser degree than when it occurred across all partnership types (Table 1).

In all scenarios that varied risk compensation by medication adherence profile and partnership types, the efficiency of PrEP (as measured by the NNT) was negatively impacted (Table 1). From the base scenario NNT of 24 under no risk compensation, the NNT reached 27, 28, 29, and 38 in the increasingly wider adherence profile scenarios in which there was 100% risk compensation. The efficiency declined because a greater amount of person-time on PrEP was required under risk compensation, since the individual-level biological prevention benefit of PrEP alone was diluted through reduced of condom use.

**Fig 2:**
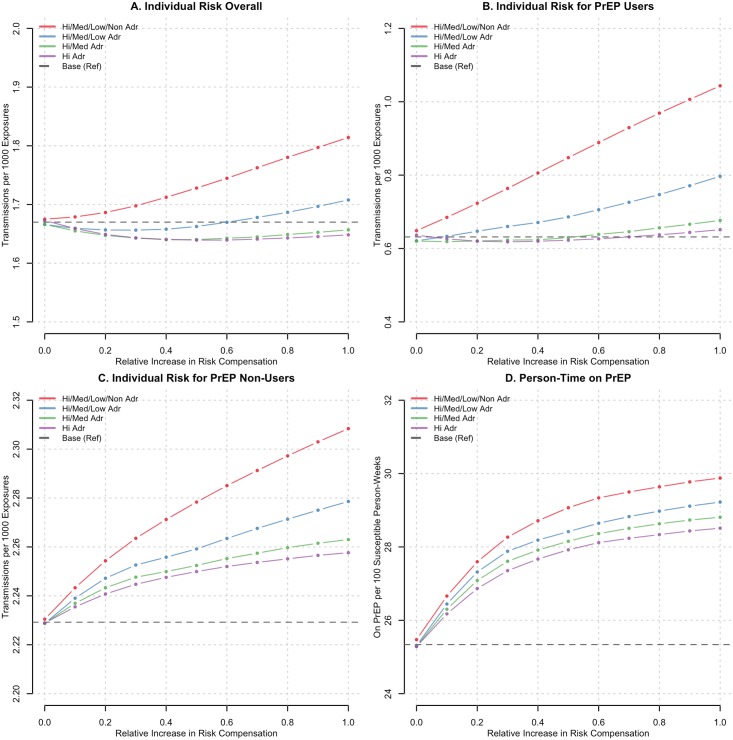
The expected rate of HIV acquisition per 1000 discordant exposures for all MSM (Panel A), and for MSM who were currently using PrEP (Panel B) or not using PrEP at the time of exposure (Panel C), by the interaction of relative levels of condom-related risk compensation (RC) and PrEP medication adherence profile in which compensation occurred. Panel D shows the amount of person-time on PrEP per 100 possible person-weeks for susceptible MSM. The red line depicts RC in all four PrEP adherence profiles (high, medium, low, and non-adherent), the blue in the top three (high, medium, low) green in the top two (high and medium) and purple in the high only. The horizontal dashed lines shows a comparison of the two outcomes (incidence and PIA) if RC were not to occur (0% RC). All points summarize the empirical distribution of 250 simulations of each scenario.

This impact on the individual-level risk is shown in Table 2 and Fig 2. In the base scenario in which there was no risk compensation, the overall individual-level risk across PrEP utilization status was 1.68 infections per 1000 exposures. The estimates for PrEP users and non-users were 0.64 and 2.23 infections per 1000 exposures, respectively. The transmission probability per 1000 exposures, regardless of current PrEP status, generally followed the same patterns as the HIV incidence rate curves in Fig 1 but on a different scale. The overall risk declined when compensation occurred within the moderately and highly adherent PrEP users (Fig 2A). However, when the transmission risk was stratified by PrEP status, it increased for both PrEP users and non-users across all adherence scenarios (Fig 2B and 2C) as risk compensation increased to 100%.

**Table 2:** Individual-Level Risk and Time on PrEP by Risk Compensation Level, by PrEP Adherence Level and Partnership Types, among US MSM.

Model Scenario	Infections per 1000 Exposures (95% CrI^1^)			Time on PrEP per 100 Person-Weeks
	*Overall*	*PrEP Users*	*PrEP Non-Users*	
**Base Scenario**				
0% RC	1.68 (1.63, 1.73)	0.64 (0.59, 0.70)	2.23 (2.16, 2.29)	25.1 (24.9, 25.4)
**Adherence Groups (All Partnership Types)**				
* High*				
50% RC	1.64 (1.59, 1.69)	0.62 (0.57, 0.67)	2.25 (2.19, 2.32)	27.9 (27.6, 28.1)
100% RC	1.65 (1.60, 1.70)	0.65 (0.61, 0.70)	2.26 (2.19, 2.33)	28.5 (28.3, 28.8)
* High*, *Moderate*				
50% RC	1.64 (1.60, 1.69)	0.64 (0.60, 0.69)	2.25 (2.18, 2.31)	28.2 (28.0, 28.4)
100% RC	1.66 (1.62, 1.70)	0.69 (0.64, 0.74)	2.26 (2.20, 2.32)	28.9 (28.7, 29.1)
* High*, *Moderate*, *Low*				
50% RC	1.66 (1.61, 1.71)	0.68 (0.64, 0.73)	2.26 (2.19, 2.33)	28.4 (28.2, 28.6)
100% RC	1.69 (1.65, 1.74)	0.77 (0.71, 0.82)	2.27 (2.20, 2.35)	29.1 (28.9, 29.4)
* High*, *Moderate*, *Low*, *Non-Adherent*				
50% RC	1.73 (1.68, 1.78)	0.85 (0.79, 0.91)	2.28 (2.22, 2.35)	29.0 (28.7, 29.2)
100% RC	1.82 (1.75, 1.87)	1.05 (0.96, 1.12)	2.31 (2.23, 2.38)	29.9 (29.7, 30.2)
**Partnership Type (All Adherence Groups)**				
* Main Partners Only*				
50% RC	1.70 (1.65, 1.74)	0.70 (0.65, 0.78)	2.23 (2.17, 2.29)	25.4 (25.0, 25.8)
100% RC	1.71 (1.66, 1.76)	0.75 (0.70, 0.82)	2.23 (2.16, 2.30)	25.4 (25.0, 25.8)
* Known HIV-discordant Partners Only*				
50% RC	1.72 (1.68, 1.78)	0.79 (0.73, 0.85)	2.23 (2.16, 2.29)	25.6 (25.1, 26.1)
100% RC	1.77 (1.73, 1.83)	0.91 (0.86, 0.98)	2.24 (2.18, 2.31)	25.7 (25.3, 26.1)

^1^ 95% CrI = 95% credible interval from the simulations

**Fig 3:**
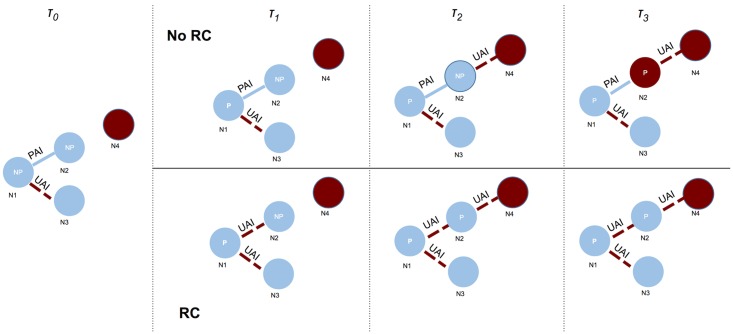
Schematic diagram showing the feedback loop between increased risk compensation, higher behavioral indications, and PrEP uptake among a four-person network over four time steps. At *t0*, Node 1 (N1) engages in condom unprotected anal intercourse (UAI) outside of a monogamous partnership (with N2 and N3), which leads to his indications for and uptake of PrEP at *t1*. In the risk compensation (RC) scenario, PrEP is associated with an increase in UAI (with N2), a PrEP non-user, whereas in the counterfactual no RC scenario, behavior remains the same as protected anal intercourse (PAI). At *t2*, N2 has engaged in UAI with N1 in the RC scenario, becomes indicated for PrEP, and uses it before initiating UAI with N4, an HIV-infected (status unknown) partner. In the *No RC* scenario, the PrEP uptake is delayed until *t3*, but N2 has already become infected by N4.

This decrease in total risk but increase in PrEP-stratified risk arose because of a unique form of dynamic confounding by indication for PrEP (Figs 2D and 3). Across all four medication adherence scenarios, risk compensation was associated with an increase in the total person-time on PrEP, with greater levels of risk compensation in wider adherence subsets associated with more PrEP utilization. This occurred because risk compensation was a change in risk behavior, and therefore the behavioral indications for PrEP, among male partners who previously had no indications. Those men engaged in more CAI with their PrEP-using partners who had reduced their condom use. Confounding occurred as more person-time was shifted from off-PrEP to on-PrEP with increasing risk compensation. This subsequently led to an overall decline in HIV incidence in those scenarios where the benefits of greater PrEP use with moderate to high adherence outweighed the increased acquisition risk from lower condom use.

## Discussion

Our model of HIV transmission dynamics suggests that condom-related risk compensation after PrEP initiation may have a minor impact on the population-level epidemiology of HIV among MSM in the US. Although potential risk compensation could simultaneously increase the individual-level acquisition risks and reduce the efficiency of PrEP, these negative outcomes would be greatest under extreme conditions of risk compensation (100% elimination of condoms during anal intercourse) and uniformity across PrEP medication adherence. Counterintuitively, when risk compensation was limited to those with moderate to high adherence to PrEP, it was associated with a decline in HIV incidence, as more men became eligible for and initiated PrEP, consequently lowering the population-level incidence rates.

Currently, few mathematical models of PrEP use among MSM have explored the phenomenon of risk compensation in this level of detail to disentangle the complex interaction between risk compensation after PrEP initiation and the behavioral indications for PrEP itself. PrEP models that incorporated risk compensation to date have suggested that negative countereffects on the ability of PrEP to prevent HIV would occur under relatively extreme conditions—just as our model required—that do not reflect current empirical data on behavioral change [14]. A recent modeling study of MSM in England simulated risk compensation as a combination of more sexual partners, less condom use, and less HIV testing after implementation of a variety of combination prevention interventions, including PrEP [30]. Although they found that HIV incidence was sensitive to some forms of risk compensation, the analysis was performed under the assumption of 100% intervention coverage, which we suggest is unrealistic for PrEP. That study also used a behavioral indication model for PrEP initiation that greatly differs from the CDC guideline representation used in our model [17,31]. Carnegie and co-authors found that PrEP prevention benefits would be mitigated only if CAI increased by 300%, requiring not only a complete replacement of condoms but also a substantial increase in new partnerships after initiating PrEP [32]. Their study also assumed lower overall adherence to PrEP medication than ours (based on iPrEx clinical trial adherence data in which the efficacy of PrEP was still unknown to study participants [1]) and did not model risk compensation as heterogeneous by adherence level, both of which could overestimate the role of risk compensation. Both our model and theirs simulated multiple types of partnerships (main versus casual). Increasing condomless anal intercourse in casual partnerships, even before the introduction of PrEP, has been an ongoing public health concern [9]. Our study suggests that the effects of risk compensation did not vary as substantially by partnership type as by adherence profile. Nevertheless, men with multiple casual partnerships are more likely to be indicated for and benefit from PrEP, and should be counseled about ongoing condom use in those partnerships in addition to PrEP use.

Ours may be the first study to explain the systems dynamics by which risk compensation fails to impact the HIV prevention benefits of PrEP. By modeling risk compensation as heterogeneous across PrEP medication adherence levels, we first observed a decline in HIV incidence when risk compensation was limited to those with moderate to high adherence levels. Understanding this counterintuitive phenomenon required further investigation of the individual-level HIV acquisition risks among PrEP users and non-users during CAI events; these risks declined overall after risk compensation was introduced but increased when stratified by PrEP use status, a novel form of dynamic and dependent confounding by indication. The confounder here was time on PrEP, which increased with higher levels of risk compensation, in an unexpected feedback loop: as more PrEP-using men engaged in risk compensation, non-PrEP using sex partners who had not previously been indicated for PrEP because they did not engage in CAI were now eligible. Fig 3 provides a schematic diagram in a four-person network to show the logic of the systems dynamics. Increased behavioral indications for PrEP led to faster rates of PrEP uptake in the model, and a greater level of protection at the community level as quantified by both acquisition risks and HIV incidence rates. These protections were offset when risk compensation occurred among low and non-adherent MSM because the increased HIV acquisition risk from non-use of condoms in those men, who received little benefit from PrEP, outweighed the increased rates of PrEP uptake.

### Limitations

This study is subject to several limitations, related to the uncertainty in long-term PrEP medication adherence, coverage, and risk compensation patterns among MSM. In particular, we modeled risk compensation as a function of the PrEP use status of one of the members in a partnership, while in reality, condom use may be a negotiated outcome between the PrEP user and his partner. Partnership-level dyadic data on the intersection of PrEP use and sexual risk behavior among MSM couples might be influential in the model, and collecting these data as PrEP uptake increases among MSM will be essential to understand the population-level implications of PrEP. Second, our results strongly depend on the relationship between behavioral indications and PrEP uptake: as PrEP indications increased with more risk compensation, so did PrEP initiation. Longitudinal data on the complex dynamics of PrEP awareness, interest, and use in varied real world settings will be critical to targeting PrEP, as well as the continued development of models like ours. Third, the generalizability of these models is partially subject to the underlying Atlanta-based behavioral data upon which the baseline models were calibrated [19,33]. As suggested in our earlier models, however, the sexual risk and HIV prevention and care access patterns are similar to national data on MSM [17]. Finally, this model did not explicitly represent non-HIV sexually transmitted infections, including gonorrhea and syphilis. Modeling this would require simulating the independent transmission dynamics of these STIs along with HIV; this is a project currently in progress.

### Conclusions

While early commentators suggested that risk compensation could be a significant vulnerability of emerging antiretroviral-based HIV prevention technologies [5,34], evidence of the phenomenon after implementation of PrEP has proven relatively limited within study settings [12–14]. It remains a central issue of discussion within the larger public health community, however, as appropriate messaging on the place of PrEP as one of several HIV prevention strategies is considered in the context of long-term patterns in sexual risk behavior among MSM [35]. Our models paint a two-sided picture: on one side, population-level epidemiology is unlikely to be impacted by risk compensation, and counterintuitively could even benefit from it under certain conditions (PrEP uptake closely follows PrEP indications); on the other, risk compensation yields this effect only because of increased PrEP utilization in the face of increased individual-level HIV acquisition risk. In situations where the supply or financial support for PrEP is below that of existing demand, this feedback loop could be diminished or eliminated, and the ability of risk compensation to increase HIV incidence may be greater. The decreased efficiency, and therefore greater associated public health expenditures, for PrEP after potential risk compensation, as well as the increased individual-level risk associated with this phenomenon, highlight the importance of promoting PrEP as a supplement rather than a replacement for condoms among MSM.
